# Interval appendectomy as a safe and feasible treatment approach after conservative treatment for appendicitis with abscess: a retrospective, single-center cohort study

**DOI:** 10.1007/s13304-023-01679-1

**Published:** 2023-11-21

**Authors:** Toshiyuki Suzuki, Akiyo Matsumoto, Takahiko Akao, Hiroshi Matsumoto

**Affiliations:** Department of Surgery, Hanyu General Hospital, Hanyushi Saitama, 348-8505 Japan

**Keywords:** Interval appendectomy, Acute appendicitis, Abscess, Conservative treatment, Emergency appendectomy

## Abstract

Emergency appendectomy (EA) is the gold standard management for acute appendicitis (AA). However, whether EA or interval appendectomy (IA) after conservative treatment is the optimal approach in AA with abscess remains controversial. This study compared IA and EA in patients presenting with AA accompanied by abscess. This was a retrospective single-center study including 446 consecutive patients undergoing appendectomy between April 2009 and March 2023. AA with abscess was defined as a pericecal abscess observed by computed tomography or abdominal ultrasonography, and patients with signs of peritoneal irritation were excluded. Perioperative outcomes were compared between the patients who directly underwent EA and those who underwent IA after conservative treatment. Among 42 patients (9.4%) with AA and abscess, 34 and 8 patients underwent IA and EA, respectively. The rates of ileocecal resection and postoperative complications were lower in the IA group than in the EA group (3% vs. 50%, *P* < 0.001 and 9% vs. 75%, *P* < 0.001, respectively). Colonoscopy before IA was performed in 16 of the 17 patients aged ≥ 40 years in the IA group, and one patient underwent ileocecal resection because of suspicious neoplasm in the root of the appendix. IA after conservative treatment might be considered as the useful therapeutic option for AA with abscess. Colonoscopy during the waiting period between the initial diagnosis and IA should be considered in patients aged ≥ 40 years who may have malignant changes. Implementing IA as a first-line treatment will be beneficial to both patients and healthcare providers.

## Introduction

Acute appendicitis (AA), i.e., inflammation of the vermiform appendix, is one of the most common causes of lower abdominal pain resulting in emergency room visits and is the most common diagnosis in younger individuals hospitalized with acute abdomen [1–3]. The estimated lifetime risk of AA is 7–8% worldwide [4]. The diagnosis of AA is based on history, physical and laboratory examination, and imaging studies, which altogether facilitate the early and accurate diagnosis of AA in more than 90% of patients [5].

AA can be classified as uncomplicated and complicated appendicitis. Uncomplicated appendicitis is not accompanied by signs of puncture, abscess, or gangrene. In contrast, complicated appendicitis is characterized by rapidly progressive gangrene, perforation, and abscess and occurs in approximately 4–25% of all patients with AA [6–10]. In patients with complicated appendicitis, emergency appendectomy (EA), which is the gold standard approach for AA [11], may increase the risk of unnecessary extended surgery, including ileocecal resection, as EA requires excessive tissue manipulation to dissect adhesions [9]. Patients with complicated appendicitis are at a higher risk postoperative intra-abdominal abscesses and surgical site infection than those with uncomplicated appendicitis. Studies show that patients with complicated appendicitis and abscesses can be effectively managed with conservative antibiotic treatment and abscess drainage followed by interval appendectomy (IA) [12, 13].

However, whether EA or IA after conservative treatment for AA with abscess is an optimal approach remains controversial [14]. In addition, few comparative studies investigated treatment strategies specifically for AA with abscess. In the present study, we aimed to elucidate optimal treatment in patients with AA accompanied by abscess, who were categorized according to the treatment strategy.

## Methods

### Patients and data collection

**Fig. 1:**
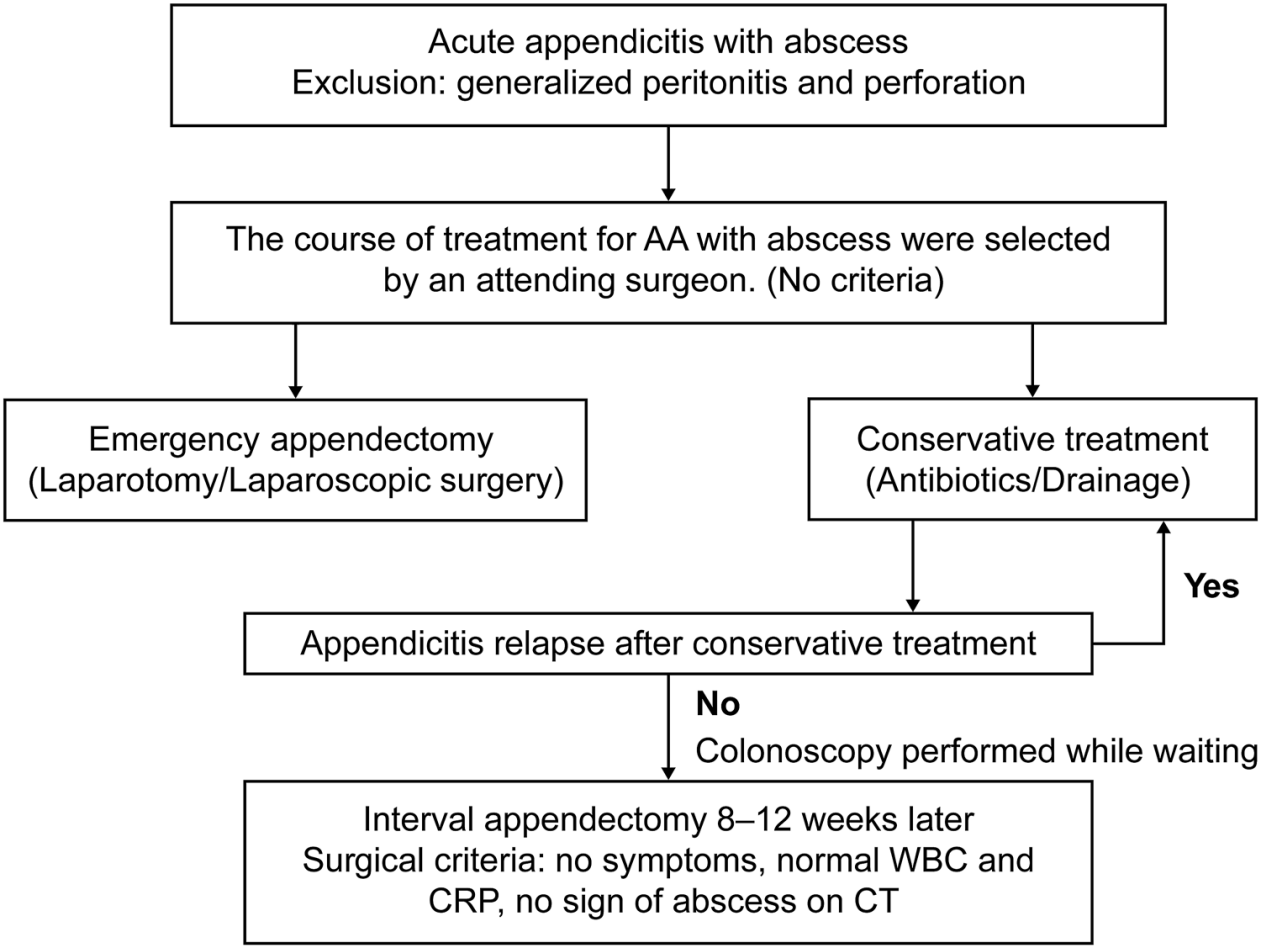
**a** Algorithm for the management of acute appendicitis with abscess. *AA* acute appendicitis, *WBC* white blood cell, *CRP* C-reactive protein, *CT* computed tomography

**Fig. 2:**
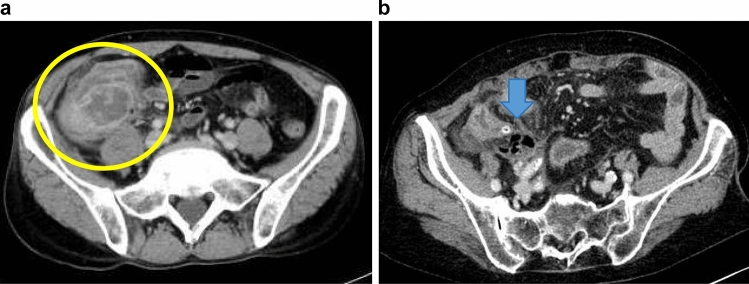
Computed tomography (CT) findings. **a** Representative case of acute appendicitis with abscess in a patient included in the study. CT image shows a pericecal abscess (yellow circle). **b** Representative case of acute perforated appendicitis in a patient excluded from the study. CT image shows free gas around the appendix (blue arrow)

A total of 446 consecutive patients underwent appendectomy at Hanyu General Hospital between April 1, 2009 and March 31, 2023. Figure 1a is an overview of the algorithm used for the management of AA with abscesses in the retrospective study hospital. In the present study, AA with abscess was defined as abscess around the appendix on computed tomography (CT) or abdominal ultrasonography in patients without any signs of peritoneal irritation (Fig. 2a). Patients with CT findings of perforation were excluded (Fig. 2b). Therefore, patients in whom CT images could not be confirmed were excluded and patients in whom an abscess was observed during the preoperative imaging of the American Association for the Surgery of Trauma Grade IV were included [15]. In the study institution, surgery was performed even in children with AA after obtaining family consent; therefore, patients aged 10 years and older were included, and no upper age limit was set. In each patient, the treatment course for AA with abscess, i.e., EA or IA, was selected at the discretion of the attending surgeon due to the lack of clear criteria. Information on patient background characteristics, perioperative data, and postoperative complications were collected from medical records. Grade II or higher complications classified as according to the Clavien–Dindo classification were included in the study.

### Surgical treatment

Laparotomy was performed through McBurney’s, paramedian, or midline incision. The incision site was selected at the discretion of the attending surgeon due to the absence of criteria. Paramedian or midline incision was chosen in cases where extended resection was possible. Appendectomy was completed by transection of the mesoappendix, followed by the ligation and resection of the appendix at the root. The appendix stump was buried with a suture, and the incision was closed in layers with or without the placement of a closed drainage tube. In cases where usual appendectomy was impossible due to inflammation, appropriate resection including ileocecal resection was performed.

During laparoscopic surgery, a 12-mm camera port was fitted into a 2–3-cm vertical skin incision in the umbilicus, and two 5-mm ports were inserted through a transverse for instruments at left abdomen [16]. The basic procedure was performed with three ports, but additional ports were inserted in patients with severe adhesion and inflammation. The appendix was ligated using an endoloop (Ethicon, Somerville, NJ, USA) or triple-row stapler (Powered Echelon Flex 60 mm; Ethicon). After placing the specimen in a collection bag, the umbilical incision was used to pull out the specimen. The procedure was switched to laparotomy in cases where laparoscopic surgery was difficult. This was mostly due to the high degree of inflammation around the cecum.

### Conservative treatment

In patients who underwent conservative treatment, intravenous or oral antibiotic treatment was administered until the resolution of fever and abdominal pain or until the return of white blood cell (WBC) count and C-reactive protein (CRP) level to near-normal levels. In cases with a large abscess around the appendix, aggressive drainage was applied. Percutaneous or transrectal drainage was selected according to the abscess location determined with CT or abdominal ultrasonography. In cases where the abscess was near the bowel and percutaneous puncture was difficult, laparotomy was selected for drainage. The drainage method was determined by the attending surgeon. In patients with relapsed appendicitis after conservative treatment, a similar conservative treatment was administered.

### IA

In cases where conservative treatment was successful, informed consent was obtained after the explanation of recurrent AA risk and IA was performed 8–12 weeks after the initial diagnosis in patients fulfilling the following conditions: (i) an interval of several months since the disappearance of symptoms, (ii) preoperative levels of WBC (< 9000/μL) and CRP (< 0.02 mg/dL) within normal range, and (iii) resolution of pericecal abscess on preoperative CT images.

At the beginning of the study, the attending surgeons chose laparotomy or laparoscopic surgery, but from the latter half of the study onwards, laparoscopic surgery was the first choice. Laparotomy or laparoscopic surgery was chosen between 2009 and 2015, whereas laparoscopic was the first choice starting in 2016. Additionally, in patients aged 40 years or older, colonoscopy was performed during the waiting period between the initial diagnosis and IA after the need for colonoscopy was explained and consent was obtained colon cancer should be ruled out. Based on the colonoscopy results, extended resection instead of appendectomy was considered.

### Statistical analysis

Patient background characteristics, perioperative data, and postoperative complications were compared between the patients who underwent EA and those who underwent IA after conservative treatment. Categorical variables were presented as numbers with percentages and compared using the chi-square test. Continuous variables were presented as medians with ranges and compared using the Mann–Whitney *U* test. A two-sided *P* value of < 0.05 was considered to indicate statistical significance. All statistical analyses were performed using JMP version 12 (SAS Institute. Cary, NC, USA).

## Results

### Patient characteristics

**Fig. 3:**
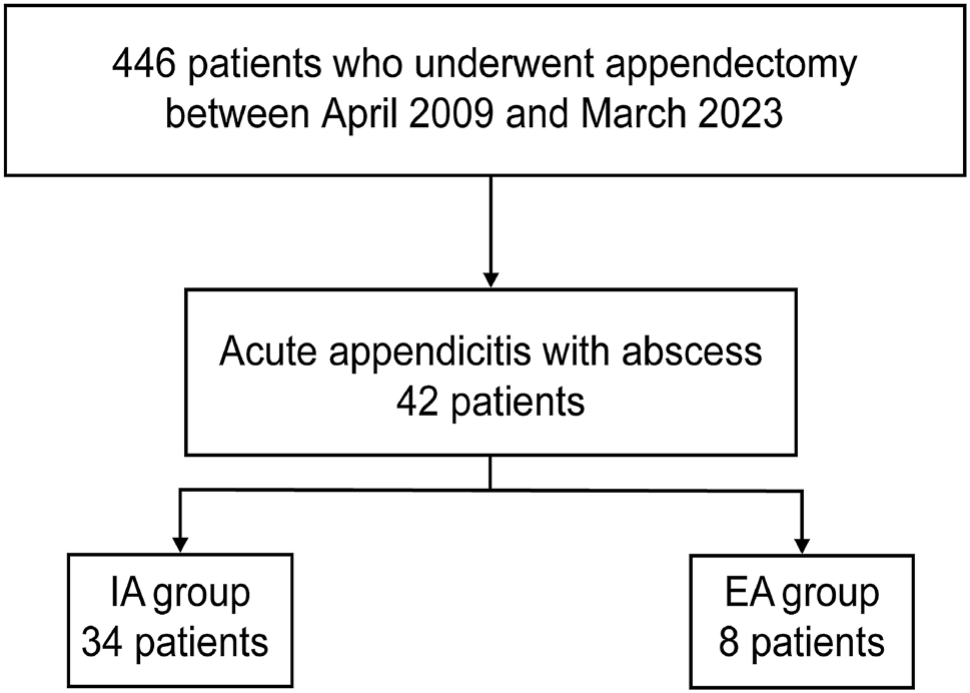
**a** Study flow diagram. *IA* interval appendectomy, *EA* emergency appendectomy

During the study period, 42 of the 446 patients who underwent appendectomy (9.4%) had AA with abscess, including 34 and 8 patients who underwent IA and EA, respectively (Fig. 3a). Table 1 shows the characteristics of patients in the IA and EA groups at their first visit. Briefly, age, median WBC count, CRP level, and maximum abscess diameter were comparable between the two treatment groups (Table 1). There was no difference in the severity of inflammation and abscess (e.g. inflammation findings and abscess diameter) between the two treatment groups.

**Table 1:** Patient characteristics

Variables	IA group (*N* = 34)	EA group (*N* = 8)	*P* value
Sex, male/female	19/15	5/3	0.734
Age, years	40 (10–80)	70 (11–87)	0.059
Period until consultation, days	6 (1–15)	7 (1–7)	0.859
BT, ℃	37.2 (36.1–39.6)	37.8 (36.1–39.2)	0.470
WBC, μL	13,135 (5030–25,070)	11,980 (5850–17800)	0.442
CRP, mg/dL	11.8 (0.04–37.6)	17.9 (4.6–26.7)	0.145
Maximum diameter of abscess, mm	36 (10–90)	39 (23–65)	0.480
Appendicolith	10 (29%)	4 (50%)	0.266

*BT* body temperature, *CRP* C-reactive protein, *EA* emergency appendectomy, *IA* interval appendectomy, *WBC* white blood cell

### Outcomes of conservative treatment

Among the 34 patients who underwent conservative treatment, 4 patients experienced exacerbation of fever and abdominal pain and underwent laparotomy (*n* = 3) and transrectal drainage (*n* = 1). In all four patients, the procedures were performed under general anesthesia. Percutaneous drainage was also performed in six patients who underwent conservative treatment. During the conservative treatment period before IA, 10 patients (29.4%) underwent drainage procedures with no associated complications. In all cases, drains were removed before IA due to reduced drainage. Abscess relapse occurred in 2 patients (5.9%), both of whom improved with conservative treatment. In all patients, the abscess eventually disappeared with successful conservative treatment.

### Preoperative management before IA

Among the 34 patients who received conservative treatment, 17 patients (50%) 40 years or older and 16 patients (94%) underwent colonoscopy while waiting for IA or had undergone colonoscopy within two years before AA. In the IA group, one patient had a tumor with findings suggesting malignancy in the root of the appendix during the waiting period; however, no indications of cancer in the cecum were found in the remaining 15 patients (94%). WBC and CRP values were within the normal range at the end of the waiting period, and the resolution of the pericecal abscess was confirmed by CT imaging. IA was performed approximately 8–12 weeks after conservative treatment in these 34 patients. The risk of occult appendiceal neoplasm increases with age, reaching a peak of 16% in patients under the age of 50, whereas the risk of AA recurrence is approximately 30%.

### Treatment course after conservative treatment in patients undergoing IA

**Fig. 4:**
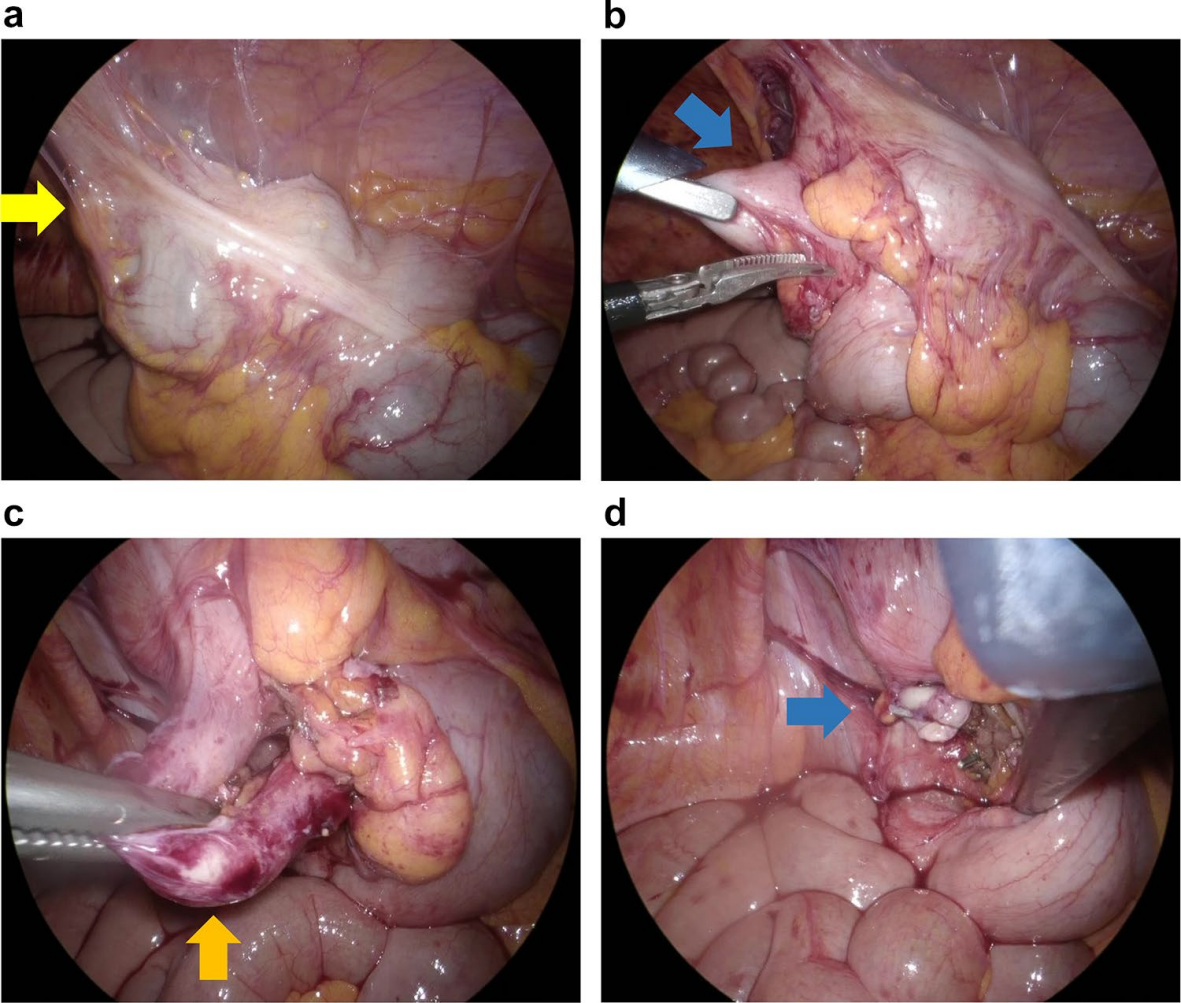
Representative intraoperative images of interval appendectomy after conservative treatment for acute appendicitis with abscess. **a** Cecum (yellow arrow) is adhering to the abdominal wall. **b** Searching for the root of the appendix (blue arrow) is feasible. **c** Adhesions around the appendix (orange arrow) are easily peeled off. **d** Resection of the root of the appendix (blue arrow) is easily achieved

The pericecal abscess diameter decreased over time with antibiotic treatment and drainage, with complete resolution observed 8–12 weeks after the initial antibiotic treatment. In patients who underwent IA, the adhesions that were usually found around the appendix were usually easy to peel off during IA (Fig. 4a, b). In all patients who underwent IA, the appendix was easily resectable at its base, without the need for extensive resection (Fig. 4c, d). In all cases, the postoperative course was uneventful and all patients were released on a median of 5 day after surgery.

### Comparison of perioperative outcomes between IA and EA

Table. 2 summarizes the perioperative outcomes of IA and EA. The duration of the operation was significantly shorter and the bleeding volume was significantly smaller in the IA group than in the EA group (47 vs. 169 min, *P* < 0.001 and 5 vs. 155 mL, *P* < 0.001, respectively). In the IA group, one patient with suspicious malignant changes on colonoscopy required ileocecal resection, which was necessary for four patients in the EA group (3% vs. 50%, *P* < 0.001). The rate of postoperative complications was significantly lower in the IA group than in the EA group (3/34 [9%] vs. 6/8 [75%], *P* < 0.001), although the total length of hospital stay (during a period of conservative management and subsequent return for IA) was not significantly different between the two group (17 and 24 days in the IA and the EA groups, respectively; *P* = 0.083). Pathologic evaluation of the resected tissue revealed tumors in three patients who underwent IA (9%), including mucinous adenocarcinoma, low-grade appendiceal mucinous neoplasm, and neuroendocrine tumor in one patient each. The patient with mucinous adenocarcinoma refused colonoscopy, which was recommended during the waiting period. The patient underwent additional resection in another hospital. In the patient with low-grade appendiceal mucinous neoplasm, the colonoscopy performed during the waiting period led to the suspicion of tumor in the root of the appendix and the patient underwent ileocecal resection instead of IA. The patient with the neuroendocrine tumor was 19 years old and did not undergo a colonoscopy during the waiting period. In contrast, the pathologic evaluation revealed inflammatory findings with no signs of malignancy in all patients who underwent EA. Ileocecal resection was performed in the EA group due to the difficulty in treating the root of the appendix.

**Table 2:** Perioperative outcomes of IA and EA

Variables	IA group (*N* = 34)	EA group (*N* = 8)	*P* value
Operating time, min	47 (22–153)	169 (93–317)	< 0.001
Bleeding volume, mL	5 (5–70)	155 (5–324)	< 0.001
*Technique*			< 0.001
Appendectomy	33 (97%)	4 (50%)	
Ileocecal resection	1 (3%)	4 (50%)	
*Operative method*			0.101
Laparotomy surgery	24 (71%)	7 (88%)	
Laparoscopic surgery	9 (26%)	0	
Conversion to laparotomy surgery	1 (3%)	1 (12%)	
Complications	3 (9%)	6 (75%)	< 0.001
SSI	3 (9%)	3 (38%)	
lleus	0	3 (38%)	
Intraperitoneal abscess	0	1 (12%)	
Other	0	2 (25%)	
Mortality	0	0	
Length of stay, day	17 (8–42)	24 (10–39)	0.083
*Pathological diagnosis*			0.661
Inflammation	32 (91%)	8 (100%)	
Mucinous adenocarcinoma	1 (3%)	0	
Low-grade apendiceal mucinous mucinous	1 (3%)	0	
NET G1	1 (3%)	0	

*EA* emergency appendectomy, *IA* interval appendectomy, *NET* Neuroendocrine tumor, *SSI* surgical site infection

## Discussion

In the present study cohort, the rate AA with abscess was 9.4%, similar to the reported rate of 2–10% [17]. Surgery was performed with fewer complications in the IA group than in the EA group, ileocecal resection, which was not needed in any patient who underwent IA, was required in some patients who underwent EA.

In previous studies, Andersson et al. reported that EA was associated with higher morbidity compared to conservative treatment (odds ratio, 3.3; 95% confidence interval, 1.9–5.6; *P* < 0.001) [9]. Simillis et al. showed that conservative treatment for complicated appendicitis was associated with lower rates of complications and reoperation compared to EA [13]. Furthermore, Miyo et al. demonstrated that single-site laparoscopic IA was a safer, more viable, and less invasive approach than EA [18]. In contrast, in a study of patients presenting with appendiceal phlegmon or abscess, Helling et al. reported that EA was preferable to conservative treatment with antibiotic administration in reducing the length of hospital stay and the need for readmissions in cases where laparoscopic expertise was available [19]. In a retrospective study by Young et al. EA was associated with superior outcomes compared to initial conservative treatment [20]. In that study including a cohort of 95 patients presenting with complicated appendicitis, 60 patients underwent EA and 35 patients initially received conservative treatment. All patients who failed conservative treatment (25.7%) underwent laparotomy, with most of the patients requiring ileocecal resection. The incidence of ileocecal resection was lower in patients who underwent EA compared to all patients who initially underwent conservative treatment (3.3% vs 17.1%, *P* = 0.048). Moreover, the Cochrane review by Cheng et al. has revealed that whether EA is superior to IA in terms of complications in patients with appendiceal phlegmon or abscess remains an unresolved question [17]. However, in these studies, the study cohorts included patients with phlegmon [9, 13, 17, 19], perforation [18], or both [20], in addition to those with abscesses. The current study findings suggest that EA is not superior to IA in patients with AA and abscess and that the findings of previous studies depend on the number of patients with AA and abscess. In the present study, IA was more effective than EA in a cohort limited to patients with AA and abscesses.

Conversely, a high-quality randomized control trial (RCT) by Mentula et al. demonstrated that laparoscopic EA was a safe and feasible first-line treatment option for AA with abscess if performed by experienced surgeons [21]. In that study, laparoscopic EA was associated with fewer readmissions (3% vs. 27%, *P* = 0.026) and fewer additional interventions (7% vs. 30%, *P* = 0.042) than conservative treatment, with a comparable length of hospital stay between the two groups. For patients in the laparoscopic EA group, the risk of ileocecal resection was 10% and the risk of incomplete appendectomy was 13%. Conversion to open surgery was necessary in 10% of the patients who underwent laparoscopic EA and in 13% of the patients who received conservative treatment [21]. The authors suggested that laparoscopic EA could be performed by experienced surgeons in the near future. On the other hand, we previously demonstrated that laparoscopic IA could aid in appendix removal and abdominal inspection [12]. Thus, we considered that implementing IA would be easier than laparoscopic EA. In addition, ileocecal resection was performed in 10% of the patients who underwent laparoscopic EA in the RCT by Mentula et al [21], whereas ileocecal resection due to the preoperative suspicion of an appendiceal tumor was performed in only one patient who underwent IA in the present study cohort. Extended resection should be avoided to the greatest extent possible in patients with benign clinical conditions such as AA. Further, the conversion rate was 3% in the current study, which was lower than the conversion rate of 10% reported in the laparoscopic EA group in the RCT. As a procedure that is less dependent on operator skills. IA might be easier and safer compared to laparoscopic EA.

Despite its success, IA after conservative treatment for AA with abscess remains a topic of debate. In cases of perforated AA and phlegmon, the recurrence rate after conservative treatment ranges from 12% to 24% [22, 23]. After initial conservative management, routine selective IA is sometimes advised to reduce the high risk of recurrence [14]. Therefore, IA was performed in all patients who underwent conservative treatment in the present study. Two of the patients (5.9%) experienced relapse after IA but improved with conservative treatment. The low recurrence rate was likely due to the fact that appendectomy was performed before recurrence in all patients who underwent IA.

Another consideration in choosing IA is the possibility of a tumor as an underlying cause of AA. Renteria et al. reported that the rate of unexpected malignancy was 3% in elderly patients with a mean age of 66 years and 1.5% in young patients with a mean age of 39 years among those who underwent appendectomy as primary treatment for AA [24]. Jonge et al. reported that appendiceal neoplasms were diagnosed in up to 11% of adult patients undergoing IA, in contrast to 1.5% of patients undergoing EA [25]. Recently, an RCT by Mällinen et al. comparing IA to follow-up with magnetic resonance imaging after initial successful conservative treatment of peri-appendicular abscess was prematurely terminated because of ethical concerns. During the interim analysis, the authors reported the unexpected finding of a high neoplasm rate (17%), with all neoplasms found in patients older than 40 years [26]. Moreover, Hayes et al. reported that the rate of appendiceal neoplasm was 11% in patients 30 years and older who underwent IA after complicated appendicitis and that the risk of appendiceal neoplasm increased with age, reaching 16% in patients 50 years and older [27]. In the present study, the neoplasm rate was 9% in the IA group, which was similar to the rate of 3%–17% reported in previous studies [24–27]. Therefore, neoplasm should be considered as a potential cause of AA with abscess and patients aged 40 years or older who present with AA by abscess should receive conservative treatment in addition to colonoscopy to rule out. In the present study, one patient underwent ileocecal resection due to the suspicion of appendiceal neoplasm based on colonoscopy. Therefore, we consider that IA is more effective than EA because it can be performed in parallel with colonoscopy during the waiting period. The presence of a tumor in one patient in the IA group may be related to chronic inflammation; however, further investigation is warranted.

In the future, IA after conservative treatment should become the first treatment option for AA with abscess and is expected to clearly reduce the rate of postoperative complications and ileocecal resection [9, 28, 29] while potentially reducing wasted medical resources. Avoiding EA in patient’s AA and abscess would also provide great benefit to healthcare providers. In addition, the possibility of a tumor can be confirmed by performing colonoscopy during the waiting period for IA, which would be beneficial to the patient.

The present study has several limitations that should be acknowledged. First, this was a retrospective, single-center study and we acknowledge the potential bias in the selection of information from the medical records. We believe that further prospective randomized multicenter investigations developed by dedicated associations and tertiary centers (high volume and strict protocols) are required to establish reliable EBM guidelines. Second, no clearly delineated criteria were utilized to determine the course of treatment for AA with abscess, which was determined by the attending surgeon treating each patient. In particular, many of the surgeons who performed the procedures included in the study could not provide a clear reason for choosing EA. Third, whether differences in the clinical course of patients were due to the involvement of different attending surgeons could not be ruled out. However, many of the attending surgeons participating in the present study were gastroenterological surgeons. In the future, to ensure the quality of surgery, it is necessary to limit the surgeons to specialists in gastroenterological surgery. Finally, laparotomy and laparoscopic approach can drastically influence the outcome of patients. The method of appendiceal root resection may also be relevant, particularly the triple-row stapler, which may be effective. However, it is unlikely that emergent surgery for appendicitis with abscess can reduce complications simply based on using a different approach or resection method. As EA crosses the abscess, residual abscess and paralytic intestinal obstruction are more likely to occur than IA.

## Conclusion

Our analyses reveal that IA after conservative treatment might be considered as the useful therapeutic option for AA with abscess based on our findings showing that EA for AA was associated with a higher rate of ileocecal resection and morbidity linked to abscess. IA after conventional therapy was associated with fewer complications and easier to perform than EA. In addition, IA is a more effective treatment option in patients aged 40 years or older who are at higher risk of exhibiting malignant change, given that IA provides an opportunity to perform colonoscopy during the waiting period between the initial diagnosis and IA. In summary, IA after conservative treatment should be considered as the first treatment option for AA with abscess, providing significant benefit to patients as well as healthcare providers.

## Data Availability

The datasets used and/or analysed during the current study are available from the corresponding author upon reasonable request.
